# Supplementary material for: Digital Assessment of the Knowledge, Attitudes and Preparedness of Dentists towards Providing Dental Treatment to People Living with HIV in Northern Brazil
Source: Int J Environ Res Public Health. 2023 Sep 27;20(19):6847. doi: 10.3390/ijerph20196847 (PMC10572858; doi:10.3390/ijerph20196847)
Supplement: Supplementary file 1 [file ijerph-20-06847-s001.zip › E-book HIV.pdf]

1ª edition

Booklet

for dental treatment of people living with HIV

Organization

Ricardo Roberto de Souza Fonseca

Paula Cristina Rodrigues Frade

Rogério Valois Laurentino

Sílvio Augusto Fernandes de Menezes

Aldemir Branco Oliveira-Filho

Luiz Fernando Almeida Machado

Collaboration

- Virology laboratory (Labvir)
- Federal University of Pará (UFPA)
- Joint United Nations Programme on HIV/AIDS (UNAIDS)
- University Center of State of Pará (CESUPA)
- Federal Council of Dentistry (CFO)
- Regional Council of Dentistry - Pará state
- Regional Council of Dentistry - Amapá state
- Regional Council of Dentistry - Amazonas state
- Regional Council of Dentistry - Rondônia state
- Regional Council of Dentistry - Roraima state
- Regional Council of Dentistry - Tocantins state
- Regional Council of Dentistry - Acre state
- Super Sorriso private dental office
- Flexa Ribeira private dental office
- University Center FIBRA (Fibra)

Summary

Preface..4
Introduction...5
Transmission pathways...............................7
HIV Window period....................................9
HIV testing...11
Signs and symptoms................................14
Oral manifestations.................................15
HIV medications.......................................20
References...23

Preface

Sexually Transmitted Infections (STIs) are mainly caused through sexual intercourse, without condoms protection or properly care, with a STI-infected sexual partner, but transmission can also occur via mother-child transmission or body fluids contact, such as blood, saliva and secretions with mucous membranes or open wounds [1].

Currently, STIs terminology should be used, mainly, among health professionals, replacing the previous term "Sexually Transmitted Diseases" (STDs), because it is understood that individuals can transmit an infection, even without showing signs and symptoms, yet, a disease is not transmissible, but the infection is [2].

According World Health Organization (WHO), it is estimated that about 376.4 million cases of curable STIs have been detected [3]. Among the main curable STIs, the following stand out: syphilis, gonorrhea, chlamydia, trichonomiasis and viral hepatitis B or C. And among the treatable STIs, but, not curable there are: herpes, HPV and HIV.

This booklet aims to set a relevant informations about HIV pathogenesis, transmission, signs and symptoms, testing, diagnosis and treatment addressed for health professionals, especially for dentists and dental workers.

Introduction

Firstly, HIV is the acronym for human immunodeficiency virus and when it infects a host, it will cause an immunosuppressive disease in the immune system and as the infection evolves, individuals may develop the acquired immunodeficiency syndrome (AIDS) [4].

This immunosuppression caused by HIV happens, because HIV infects immune system cells, such as neutrophils, macrophages, dendritic cells and CD4+ T lymphocytes.

HIV is classified as a retrovirus genus, *Lentiviridae* subfamily, has genetic material composed by RNA and has two species: HIV-1 of worldwide prevalence and HIV-2 found in certain regions such as West Africa [5].

LabVir
Laboratório de Virologia ICB/UFPA

Introduction

Figure 1: **HIV-1** illustrative image

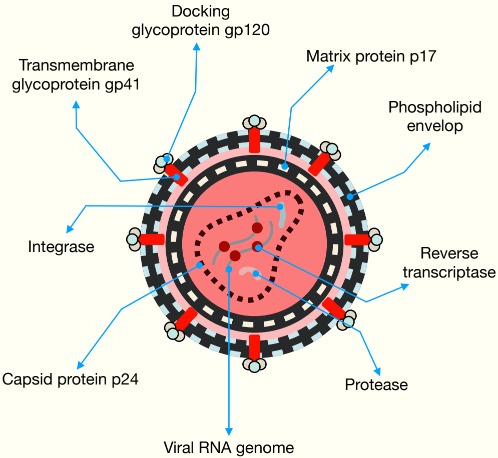

Source: Fonseca et al. 2023

Transmission pathways

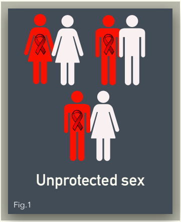

Fig.1

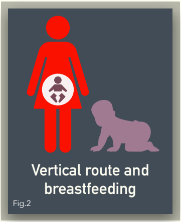

Fig.2

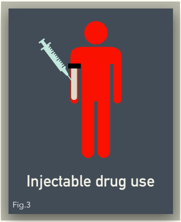

Fig.3

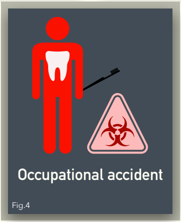

Fig.4

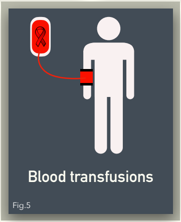

Fig.5

Incorrectly, personal contact (hand shaking or hugs), insect bites, sharing personal hygiene items (soap) or sharing food items (cutlery and cups) and via aerosol suspension during dental care [6], are, popularly, considered HIV transmission pathways, **however**, literature points out that transmission pathways mentioned above **are not correctly**, **just social stigmas** [7].

Source: Fonseca et al. 2023

Window
period

For dentistry professionals, it is important to know about HIV window period. **Window period is time interval between** HIV initial infection to first signs of HIV infection and the first anti-HIV antibodies detection produced by the host's immune system, it is worth mentioning that even in this window period **HIV can be transmitted** [8].

Generally, common window period duration is about **30 days**, after initial risk situation and transmission, due to viral replication cycle inside the cells. However, window period duration can vary by up to **90 days**, depending on HIV-infected individual systemic conditions [9].

Therefore, while testing for anti-HIV antibodies detection, HIV window period must be considered in order the possibility of **non-reactive result**, even when individual is already infected. Thus, it is recommended that during HIV window period, if individuals present a **non-reactive results**, although individuals **presents HIV infection suspicious**, testing must be repeated for 90 days.

How window period works

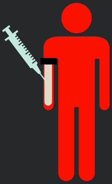

Fig 1. Transmission by injectable drug use

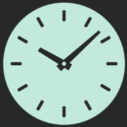

Fig 2. HIV viral replication period

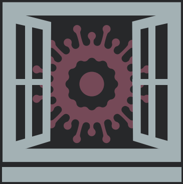

Fig 3. HIV window period

Source: Fonseca et al. 2023

HIV testing
methods

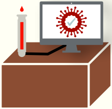

Laboratory tests for HIV **diagnostic purposes** can be performed from oral fluid, serum, plasma or whole blood collection and conducted by **Enzyme Immunoabsorption Test (ELISA)**, which has the function of immunoglobulin G (IgG) detecting, it is used in laboratory diagnostic routine and **western blot type** is the gold standard for HIV diagnosis. Elisa interpretation is very simplified, results can be: non-reactive; reactive and indeterminate.

It is important to emphasize that there are different tests types for different stages of HIV infection and all can be ordered by dentists. Longer laboratory tests as ELISA and rapid tests, in Brazil, are performed charge free at **Unified Health System (SUS)** and at **Testing and Reception Center (CTA)**, which are specialized centers in counseling, diagnosis and treatment of HIV, viral hepatitis, syphilis and various STIs [10].

Rapid tests are kits recommended for face-to-face testing, commonly, used after occupational accidents, like dental care. Rapid tests are practical and can be carried out in up to 30 minutes, which after material collection, it must be put in contact with reagents in an eppendorf and wait the time indicated by manufacturer, and the results may be non-reactive; reactive and indeterminate [11].

Source: Fonseca et al. 2023

HIV testing
ELISA

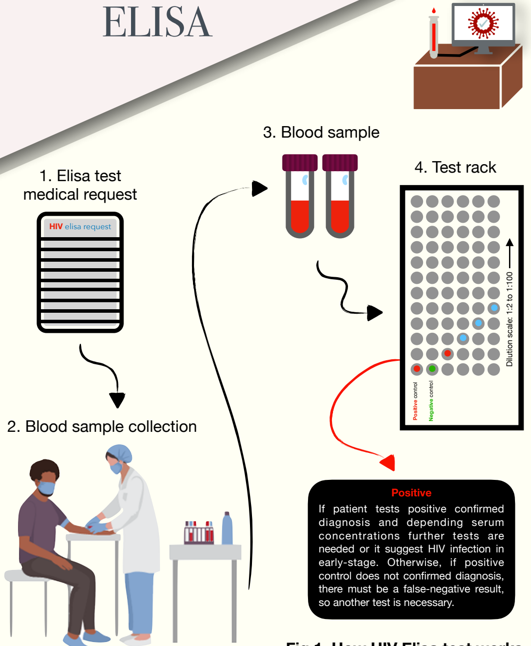

Source: Centers for Disease Control and Prevention

Fig 1. How HIV Elisa test works

Source: Fonseca et al. 2023

HIV testing
rapid

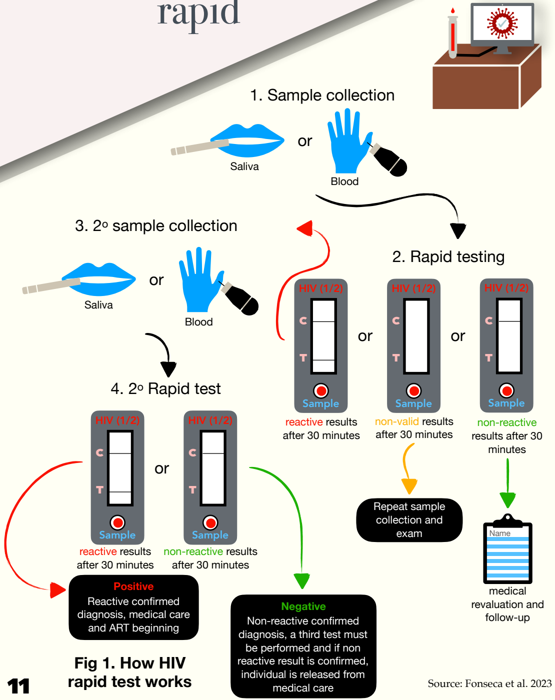

Fig 1. How HIV rapid test works

Source: Fonseca et al. 2023

Testagem

T CD4 +

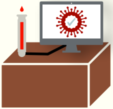

In confirmed HIV-infected patients, dentists and other health professionals may request CD4+ T cell count tests and viral load quantification, which are used to monitor HIV infection and monitoring antiretroviral therapy (ART) efficiency or even ARTviral resistance. CD4+ T monitoring consists of the absolute count of CD4+ T lymphocytes (mm3) in blood or saliva, this test is used as a laboratory parameter for clinical levels of immune system cells, associated with leukogram, erythrogram and coagulogram, which predicts HIV infection course , directing whether the patient is in the acute, chronic or AIDS phase, otherwise is also to identify possible opportunistic infections, [11].

**CD4+ T cell counting**
**HIV infection absence: Individual will have a rate of 500 to 1200 copies of CD4+ T lymphocytes/mm3 in blood**
**Individual living with HIV in regular ART: usually has ≥ 350c/mm3 in blood**
**Individual living with HIV in irregular ART: usually has ≤ 350c/mm3 in blood**
**Individual living with untreated HIV: usually has ≤ 200c/mm3 in blood and a high risk of AIDS and opportunistic infections**

Table 1: CD4+ T cell count clinical parameters, another form of analysis is by percentage which ≥ 29% T CD4+ cells is = a ≥ 500c/mm3/ ≤ 14% T CD4+ cells is = a ≤ 200c/mm3.

Source: Fonseca et al. 2023

Testagem

Carga viral

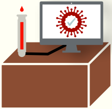

Viral load quantification seeks to determine the amount of viral RNA particles produced and released into the bloodstream. Commonly, in HIV infection cycle, during acute phase, viral particles counting is higher and lower in chronic phase, and there may be an exponential increase during AIDS [10].

Therefore, there is a proportional direct relationship between amount of viral particles detected and HIV infection progression/morbidity. So, patients with high viral load and low levels of CD4+ T cells has an accelerated deterioration of immune system, as well as a higher risk of HIV transmission, also to transmit secondary infections, such as, tuberculosis or cytomegalovirus mononucleosis [11].

Viral load quantification
100.000 to 1.000.000 viral copies/mm3 in blood is considered a high level of viral load
≥ 10.000 viral copies/mm3 in blood indicates individuals living with HIV without adequate treatment and at transmission risk
≤ 10.000 viral copies per mm3 in blood indicates individuals living with HIV with regular treatment and no risk of transmission
Individual living with untreated HIV: usually has ≤ 200/mm3 in blood and is at high risk of developing AIDS and opportunistic infections
≤ 50 viral copies/mm3 in blood or "undetectable" viral load is the goal of regular ART and there is no risk of transmission

Table 2: Viral load clinical parameters.

Source: Fonseca et al. 2023

SYSTEMIC

Symptoms signs

Clinically, after contamination, certain signs and symptoms of infection will appear within a few days to weeks from contamination and, generally, the first signs and symptoms observed are very similar to those of a prolonged common flu or mononucleosis [12].

Clinical characteristics are heterogeneous, that is, HIV-infected individuals may vary between signs, and symptoms depending on HIV infection stages. During HIV infection stages, people living with HIV (PLWH) may remain without symptoms, but transmissible. Therefore, individuals who delay treatment star or are inconsistency in it, may aggravate the infection due to systemic opportunistic infections such as: viral hepatitis, tuberculosis and pneumonia, as well as presenting oral manifestations [13].

1. Fever
2. Rash and chills
3. Nausea
4. Appetite loss
5. Constant diarrhea
6. Weight loss
7. Swollen lymph nodes
8. Excessive fatigue
9. Muscle aches
10. Night sweats
11. Arthritis
12. Sore throat

Oral manifestations

Oral manifestations or lesions manifestations are among the first indicators of a possible HIV infection, therefore, dentists and dental professionals are fundamental in diagnosis and treatment of HIV, mainly being familiar with signs and symptoms, transmission pathways, HIV infection cycle and currently therapies aimed to treat HIV [14,15].

Unfortunately, studies show that dentists are not very well familiar with specific knowledges about HIV, which can lead to certain stigmas or fears when treating PLWh, as shown in the literature about 45.1% among 51 dentists make changes to their non-invasive dental care procedures, when known that patient is HIV-infected. Also, about 31.4% among 51 dentists make changes to their invasive dental care procedures, when known that patient is HIV-infected [6,15].

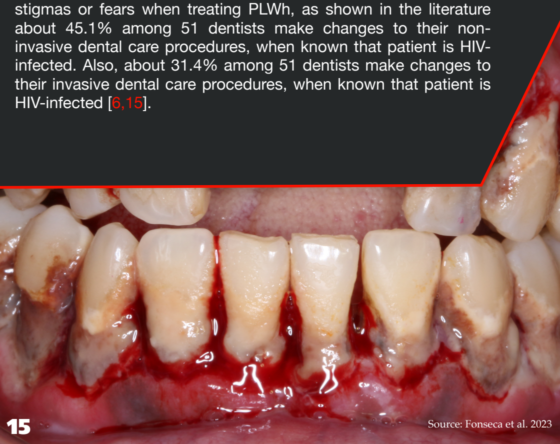

Source: Fonseca et al. 2023

Oral manifestations

Fonseca et al. demonstrated that about 82.4% were unaware of the most common oral lesions of PLWH, which demonstrates a worrying sign for such relevant professionals, since oral lesions can occur in up to 50% of PLWH and in up to 80% with AIDS, and oral lesions presence associated with HIV has a great negative impact on quality life of PLWH, favoring malnutrition, immunosuppression, increased virulence and morbidity [6,15].

These results reveal a certain lack of knowledge regarding common oral lesions in PLWH. Therefore, it is necessary to disseminate this knowledge among dentists, dental students and dental professionals. For more information on PLWH oral lesions visit: https://www.intechopen.com/chapters/84208

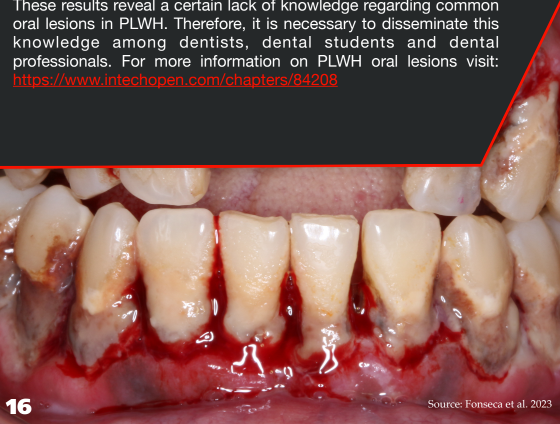

Source: Fonseca et al. 2023

Oral manifestations

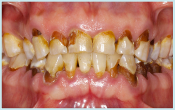

Fig 1. **Dental cavity**
(Source: Lomelí-Martínez et al. 2022)

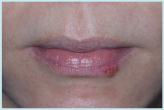

Fig 2. **Labial herpes**
(Source: Fonseca et al. 2023)

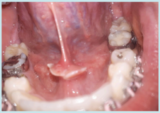

Fig 3. **Oral Warts**
(Source: Lomelí-Martínez et al. 2022)

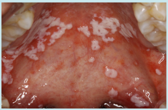

Fig 4. **Erythematous candidiasis**
(Source: Lomelí-Martínez et al. 2022)

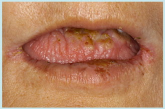

Fig 5. **Xerostomia**
(Source: Lomelí-Martínez et al. 2022)

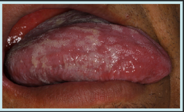

Fig 6. Oral Hairy Leukoplakia
(Source: Lomelí-Martínez et al. 2022)

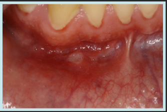

Fig 7. Aphthous Ulcers
(Source: Lomelí-Martínez et al. 2022)

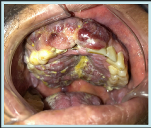

Fig 8. Kaposi's sarcoma
(Source: Lomelí-Martínez et al. 2022)

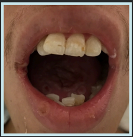

Fig 9. Angular cheilitis
(Source: Lomelí-Martínez et al. 2022)

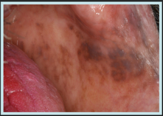

Fig 10. Oral Hyperpigmentation
(Source: Lomelí-Martínez et al. 2022)

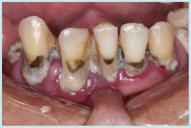

Fig 11. Periodontitis
(Source: Fonseca et al. 2023)

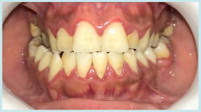

Fig 12. Linear gingival erythema
(Source: Fonseca et al. 2023)

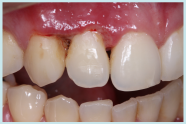

Fig 13. Necrotizing gingivitis
(Source: Fonseca et al. 2023)

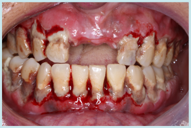

Fig 14. Necrotizing periodontitis
(Source: Fonseca et al. 2023)

HIV medicines

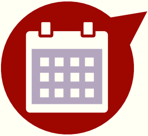

HIV ART emerged in the 1980s, these medications intended to prevent HIV progression through immune system and cause immunosuppression among key populations [16].

It is important to emphasize that ART cannot totally eliminate HIV, but ART medications were designed to act in several stages of HIV cycle, in order to control virus replication and avoid severe suppression of the immune system [17].

Nucleotide Reverse Transcriptase Inhibitors (NRTIs)

Inhibit HIV reverse transcriptase enzyme and operate by incorporation into viral DNA.

Non-nucleoside Reverse Transcriptase Inhibitors (NNRTIs)

Induce allosteric changes, rendering the enzyme incapable of converting viral RNA to DNA.

Protease Inhibitors (PIs)

Protease inhibitors block HIV maturation

Integrase Inhibitors

Block retroviral integration from catalyze within a nucleoprotein.

Capsid Inhibitor

Disrupt HIV capsid during multiple stages of viral life cycle.

Fusion Inhibitors

Block fusion between HIV to human CD4 cell reducing HIV entry into cells.

gp120 Attachment Inhibitor

Interfere with the interaction between the gp120 protein on HIV outer surface and CD4 cell receptor.

CCR5 Antagonist

Block CCR5 coreceptor on the surface of CD4 cells and others immune cells from link to HIV.

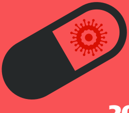

HIV medicines

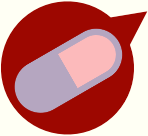

ART, when prescribed, aims to reduce viral RNA plasma levels down to undetectable levels (<20 to 50 copies/mL) and maintain the CD4 cells counting at between 500 to 1500 cells/mm^3, thus reducing morbidity, mortality, increasing quality of life of PLWH and life expectancy [17].

Therefore, nowadays it is understood that HIV seropositive terms are stigmatizing and offensive to PLWH, especially when used by health professionals such as dentists [18].

Currently, Joint United Nations Program on HIV/AIDS (UNAIDS), acts to create solutions in HIV and AIDS combat, as well as, offer treatment or assistance to HIV-infected people, for the purpose to improve PLWH of quality of life, moreover we should use terms like people living with HIV or HIV/AIDS when referring to people who are infected with HIV as a way to decrease stigma and prejudice towards PLWH [19].

ART use is fundamental for HIV treatment and since 1996, in Brazil, SUS distributes a drugs free cocktail, popular known as three in one, because it is composed of 3 different types of ART's in an attempt to act at different stages of viral cycle: Tenofovir (300mg), Lamivudine (300mg) and Efavirenz (600mg) [20].

Source: Fonseca et al. 2023

HIV medicines

PrEP

Pre-exposure prophylaxis (PrEP) refers to antiretroviral medicine prescribed before exposure (or possible exposure) to HIV [21-23].

PrEP is indicated for individuals belonging to key populations to HIV and a daily oral dose of ART reduces the risk of HIV transmission [21-23].

PEP

Post-exposure prophylaxis (PEP) refers to medicine used for cases after exposure or possible exposure to HIV. Exposure cases can be occupational or non-occupational [21-23].

PEP must be used within 72 hours after exposure and during for 28 consecutive days, also if started within the first 2 hours after exposure, effectiveness is higher [21-23].

Source: Fonseca et al. 2023

References

1. Domingues CSB, Lannoy LH, Saraceni V, Cunha ARCD, Pereira GFM. Brazilian Protocol for Sexually Transmitted Infections 2020: epidemiological surveillance. Rev Soc Bras Med Trop. 2021, 54(suppl 1): e2020549.
2. Miranda AE, Freitas FLS, Passos MRL, Lopez MAA, Pereira GFM. Public policies on sexually transmitted infections in Brazil. Rev Soc Bras Med Trop. 2021, 54(suppl 1):e2020611.
3. World Health Organization (WHO). Sexually transmitted infections [Internet]. 2022. Available online: https://www.who.int/news-room/fact-sheets/detail/sexually-transmitted-infections-(stis). (Accessed in February 23th 2023).
4. World Health Organization (WHO). HIV/AIDS topics [Internet]. 2022. Available online: https://www.who.int/health-topics/hiv-aids#tab=tab_1. (Accessed in February 23th 2023).
5. Machado LFA, Fonseca RRS, Queiroz MAF, Oliveira-Filho AB, Cayres-Vallinoto IMV, Vallinoto ACR, et al. The Epidemiological Impact of STIs among General and Vulnerable Populations of the Amazon Region of Brazil: 30 years of Surveillance. Viruses. 2021, 13(5):855.
6. de Souza Fonseca RR, Laurentino RV, de Menezes SAF, Oliveira-Filho AB, Alves ACBA, Frade PCR, et al. Digital Form for Assessing Dentists' Knowledge about Oral Care of People Living with HIV. Int J Environ Res Public Health. 2022, 19(9): 5055.
7. Levy JA. Pathogenesis of human immunodeficiency virus infection. Microbiol Rev. 1993, 57(1):183-289.
8. Brazilian Ministry of Health (BMH). AIDS/HIV topics [Internet]. 2021. Available online: https://www.gov.br/saude/pt-br/assuntos/saude-de-a-a-z/a/aids-hiv. (Accessed in March 1st 2023).
9. Fonseca RRS, Laurentino RV, Machado LFA, Gomes CEVS, Menezes TOA, Faciola OFP, Oliveira-Filho AB, et al. HIV Infection and Oral Manifestations: An Update. Future Opportunities and Tools for Emerging Challenges for HIV/AIDS Control [Internet]. 2023, Feb 8th; Available online: https://www.intechopen.com/chapters/84208.

References

10. Brazilian Ministry of Health (BMH). AIDS diagnosis [Internet]. 2022. Available online: https://www.gov.br/saude/pt-br/assuntos/saude-de-a-a-z/a/aids-hiv/diagnostico-da-aids-hiv. (Accessed in March 10th 2023).
11. Brazilian Ministry of Health (BMH). CD4+ counting and viral load [Internet]. 2022. Available online: https://bvsms.saude.gov.br/bvs/publicacoes/16contagem_celulasTCDA.pdf. (Accessed in March 10th 2023).
12. Menezes TO, Rodrigues MC, Nogueira BM, Menezes SA, Silva SH, Vallinoto AC. Oral and systemic manifestations in HIV-1 patients. Rev Soc Bras Med Trop. 2015, 48(1):83-6.
13. Heron SE, Elahi S. HIV Infection and Compromised Mucosal Immunity: Oral Manifestations and Systemic Inflammation. Front Immunol. 2017, 7;8:241.
14. Lomelí-Martínez SM, González-Hernández LA, Ruiz-Anaya AJ, Lomelí-Martínez MA, Martínez-Salazar SY, Mercado González AE, et al. Oral Manifestations Associated with HIV/AIDS Patients. Medicina (Kaunas). 2022, 58(9):1214.
15. da Rocha GCT, Fonseca RRS, Oliveira-Filho AB, Ribeiro ALR, de Menezes SAF, Laurentino RV, et al. Evaluation of Sociodemographic Factors and Prevalence of Oral Lesions in People Living with HIV from Cacoal, Rondônia, Amazon Region of Brazil. Int J Environ Res Public Health. 2022, 19(5):2614.
16. Oswaldo Cruz Foundation (Fiocruz). Antiretrovirals through history, from discovery to present day [Internet]. 2011. Available online: https://www.arca.fiocruz.br/handle/icict/11130. (Accessed in March 18th 2023).
17. Joint United Nations Programme on HIV/AIDS (UNAIDS). Important definitions [Internet]. 2011. Available online: https://unaids.org.br/desafiounaids/index.php/manual/definicoes-importantes/. (Accessed in March 18th 2023).

References

18. Joint United Nations Programme on HIV/AIDS (UNAIDS). Antiretrovirals [Internet]. 2015. Available online: https://unaids.org.br/2015/02/estudos-mostram-eficacia-de-antirretrovirais-na-prevencao-ao-hiv/ Estudos mostram eficácia de antirretrovirais na prevenção ao HIV. (Accessed in March 18th 2023).
19. Joint United Nations Programme on HIV/AIDS (UNAIDS). Antiretrovirals treatment [Internet]. 2015. Available online: https://unaids.org.br/tag/tratamento-antirretroviral/ OMS recomenda o dolutegravir como principal opção de tratamento para o HIV em todas as populações. (Accessed in March 18th 2023).
20. Brazilian Ministry of Health (BMH). STI's pharmaceutical protocol [Internet]. 2010. Available online: https://bvsms.saude.gov.br/bvs/publicacoes/protocolo_assistencia_farmaceutica_aids.pdf protocolo de assistência farmacêutica em dst/hiv/aids. (Accessed in March 18th 2023).
21. Sousa LRM, Elias HC, Fernandes NM, Gir E, Reis RK. Knowledge of PEP and PrEP among people living with HIV/aids in Brazil. BMC Public Health. 2021, 21(1):64.
22. Grangeiro A, Couto MT, Peres MF, Luiz O, Zucchi EM, de Castilho EA, et al. Pre-exposure and postexposure prophylaxes and the combination HIV prevention methods (The Combine! Study): protocol for a pragmatic clinical trial at public healthcare clinics in Brazil. BMJ Open. 2015, 5(8):e009021.
23. Maksud I, Fernandes NM, Filgueiras SL. Technologies for HIV prevention and care: challenges for health services. Rev Bras Epidemiol. 2015, 18 Suppl 1:104-19.
